# Expression of acid cleavable Asp-Pro linked multimeric AFP peptide in *E. coli*

**DOI:** 10.1186/s43141-021-00265-5

**Published:** 2021-10-14

**Authors:** Murad Mollaev, Artur Zabolotskii, Neonila Gorokhovets, Elena Nikolskaya, Maria Sokol, Andrey Tsedilin, Mariia Mollaeva, Margarita Chirkina, Timofey Kuvaev, Anna Pshenichnikova, Nikita Yabbarov

**Affiliations:** 1 Biotechnology and Industrial Pharmacy Department, Lomonosov Institute of Fine Chemical Technologies, MIREA – Russian Technological University, 86 Vernadsky avenue, Moscow, 119454 Russia; 2 Dmitry Rogachev National Medical Research Center of Pediatric Hematology, Oncology and Immunology, Laboratory of Molecular Immunology, 1 Samory Mashela street, Moscow, 117997 Russia; 3 JSC Russian Research Center for Molecular Diagnostics and Therapy, 8 Simferopolsky boulevard, Moscow, 117638 Russia; 4 Department of Biochemistry, Biological Faculty, Lomonosov Moscow State University, 1-12 Leninskie Gory, Moscow, 119991 Russia; 5 I.M. Sechenov First Moscow State Medical University, 8-2 Trubetskaya street, Moscow, 119991 Russia; 6 N. M. Emanuel Institute of Biochemical Physics, RAS. 4 Kosygina street, Moscow, 119334 Russia; 7 Fundamentals of Biotechnology Federal Research Center, RAS, 33 Leninsky avenue, Moscow, 119071 Russia; 8 National Research Center “Kurchatov Institute”, Research Institute for Genetics and Selection of Industrial Microorganisms, 1 1-Y Dorozhnyy Proyezd, Moscow, 117545 Russia

**Keywords:** Multimer expression, Asp-Pro cleavage, Alpha-fetoprotein, Recombinant peptide

## Abstract

**Background:**

Difficult to express peptides are usually produced by co-expression with fusion partners. In this case, a significant mass part of the recombinant product falls on the subsequently removed fusion partner. On the other hand, multimerization of peptides is known to improve its proteolytic stability in *E. coli* due to the inclusion of body formation, which is sequence specific. Thereby, the peptide itself may serve as a fusion partner and one may produce more than one mole of the desired product per mole of fusion protein. This paper proposes a method for multimeric production of a human alpha-fetoprotein fragment with optimized multimer design and processing. This fragment may further find its application in the cytotoxic drug delivery field or as an inhibitor of endogenous alpha-fetoprotein.

**Results:**

Multimerization of the extended alpha-fetoprotein receptor-binding peptide improved its stability in *E. coli*, and pentamer was found to be the largest stable with the highest expression level. As high as 10 aspartate-proline bonds used to separate peptide repeats were easily hydrolyzed in optimized formic acid-based conditions with 100% multimer conversion. The major product was represented by unaltered functional alpha-fetoprotein fragment while most side-products were its formyl-Pro, formyl-Tyr, and formyl-Lys derivatives. Single-step semi-preparative RP-HPLC was enough to separate unaltered peptide from the hydrolysis mixture.

**Conclusions:**

A recombinant peptide derived from human alpha-fetoprotein can be produced via multimerization with subsequent formic acid hydrolysis and RP-HPLC purification. The reported procedure is characterized by the lower reagent cost in comparison with enzymatic hydrolysis of peptide fusions and solid-phase synthesis. This method may be adopted for different peptide expression, especially with low amino and hydroxy side chain content.

**Supplementary Information:**

The online version contains supplementary material available at 10.1186/s43141-021-00265-5.

## Background

Recombinant peptide expression is an alternative to solid-phase synthesis. Relatively short polypeptides are difficult to express in *E. coli*, which is probably due to the active proteolysis of cytosolic soluble products [1, 2]. The common way to improve the stability of peptides in the cytosol is to co-express peptides with fusion partners such as PurF fragment, ketosteroid isomerase, PaP3, and TAF12 domain. Moreover, the fusion partner can be the same peptide itself, when expressed in tandem repeats [3–5]. Multimeric expression as well as fusion co-expression requires the specific enzymatic or chemical cleavage site(s). Enzymatic digestion with factor Xa (IEGR↓), enterokinase (DDDDK↓), thrombin (LVPR↓GS), TEV protease (ENLYFQ↓G), and HRV 3C protease (LEVLFQ↓GP) is the most common methods for hydrolysis of fusion proteins [6–9]. These methods are highly specific, but limited to enzyme costs and sensitivity to environmental changes. Denaturing conditions can be detrimental to enzyme activity, while most peptide multimers tend to form aggregates. Alternatively, chemical cleavage has lower reagent costs, wider temperature and suitable pH range, solubilization agent compatibility, and shorter artificial sequence insertions [10, 11]. Some peptide bonds are known to be less acid stable than others. Acid lability of –D–X– bond (where X = any amino acid) was discovered by Partridge and Davis [12], but the technique was of little use due to the extremely critical conditions of such cleavage. Among –D–X– bonds, –D–P– and –D–C– were found to be the most acid sensitive [13, 14]. The mechanism of –D–P– bond acid hydrolysis is that the N atom of proline attacks protonated sidechain carboxyl of aspartate, thus forming an unstable cation-imide intermediate, which is then become rapidly hydrolyzed. Due to the lower abundance of –D–P– bonds, this technique has an advantage over BrCN cleavage [15]. The use of such expression system has great prospects in the production of low molecular weight peptides for targeted therapy (highly specific inhibitors or agonists, vector molecules, etc.), since the low molecular weight peptides tend to be more proteolytically stable in multimers and have a low frequency of –D–P– bonds. Human AFP is an albumin family protein that primarily functions as a transporter of lipophilic molecules and several ions during the fetal period. AFP is capable of triggering various signaling pathways, including those that promote immunosuppression [16]. The expression level of AFP in healthy adults is significantly lower than in the fetal period; however, AFP is detected in patients with hepatocellular carcinoma and some other cancers [17]. AFP is known to selectively bind and to be internalized by a wide range of cancer cells. Different researchers reported its suitability for anticancer drug delivery systems as targeting motif [18–21]. Recent studies showed that endogenous full-length AFP may promote hepatic cancer progression and is not recommended for human treatment; however, the mechanism is not completely understood [22, 23]. Shorter functional fragments may still prove its safety [22], while remaining specific activity. Arguably, short fragments may block epitopes recognized by native endogenous AFP, thereby inhibiting its immunosuppressive and tumor-stimulating functions. Previously, the KQEFLIN peptide was found to be the minimal and necessary AFP part for receptor binding [24]. Here, we report the multimeric –D–P– linked extended AFP receptor-binding fragment expression with optimized multimerization, cleavage, and purification conditions.

## Methods

The reagents used in this study are provided in Supplementary information 2.

### Cloning and expression

Molecular cloning was performed by the standard procedures [25]. Plasmid DNA was extracted using the Quantum Prep Plasmid Miniprep Kit. In order to amplify the sequence coding, PWGVALQTMKQEFLINLVKQKPQITD peptide A-C primers have been selected according to the human AFP mRNA (GenBank NM_001134): (A) ATTCCATGGCTGATCCGTGGGGTGTAGCG (NcoI restriction site is underlined); (B) ATTGTCGACGATCCGTGGGGTGTAGCGC (SalI restriction site is underlined); (C) ATATCTCGAGCGGATCTGTAATTTGTGGC (XhoI restriction site is underlined). Insertion 1 (supplementary information Fig. S1) was amplified with the forward primer A and reverse primer C and with the previously designed plasmid, encoding C-end domain of AFP [26] as a matrix. Insertion 2 (supplementary information Fig. S1) was prepared in the same way by using primers B and C. Insertion 1 was cloned into pET-28a(+) expression vector using NcoI and XhoI. The resulting plasmid pET28AFPpep1 encoded monomeric peptide. To obtain plasmids encoding different numbers of tandem repeats (from 2 to 14), Insertion 2 was digested with SalI and XhoI and sequentially cloned into the XhoI site of pET28AFPpep1. The number of inserts was analyzed by PCR with T7 promotor and terminal primers followed by agarose electrophoresis. To ensure the forward direction of all inserts, PCR with T7 terminal and C reverse primers was used.

Constructed pET28AFPpepN plasmids were transfected into *E. coli* BL21(DE3) expression strain (NEB, USA). Clones were selected with kanamycin (50 μg/ml) on agarized LB plates. One colony for each sample was inoculated into a 5-ml LB medium with kanamycin (50 μg/ml) and cultured overnight at 37°C with shaking. Further cells were inoculated into fresh LB medium in ratio 1:50 vol. and cultured at 37°C with shaking until OD600 reached 0.8. The expression of pET28AFPpepN was induced by 1 mM IPTG for 4 h. For expression analysis, 50-μl samples were taken at each stage and analyzed by SDS-PAGE.

### Isolation and purification of multimeric peptide

Multimeric peptide AFPpep5 (MA**DPW**GVALQTM*KQEFLIN*LVKQKPQIT**DP**LD**DPW**GVALQTM*KQEFLIN*LVKQKPQIT**DP**LD**DPW**GVALQTM*KQEFLIN*LVKQKPQIT**DP**LD**DPW**GVALQTM*KQEFLIN*LVKQKPQIT**DP**LD**DPW**GVALQTM*KQEFLIN*LVKQKPQIT**DP**LEHHHHH) was purified by immobilized metal affinity chromatography on Ni^2+^ Sepahrose 6 Fast Flow (GE Healthcare). For this purpose, 40 ml of Ni^2+^ Sepahrose 6 Fast Flow was packed into a 50 × 150 column and equilibrated with binding buffer (50 mM Tris-HCl pH 7.5, 6 M guanidine hydrochloride). The wet biomass was resuspended in 100 ml of 50 mM Tris-HCl buffer (pH 7.5) on ice and sonicated 3 times, 40 strokes each. The suspension was centrifuged at 10,000g, 4°C for 15 min. The precipitate containing inclusion bodies was thrice resuspended in the same buffer and centrifuged. Washed precipitate was dissolved in 100 ml of 50 mM Tris-HCl buffer (pH 7.5) containing 6 M guanidine hydrochloride and centrifuged at 10,000 rpm (4 °C, 15 min). Solubilized inclusion bodies (6 g) from the supernatant were applied to the column, followed by washing with 3 volumes of binding buffer. Multimer was eluted by 1 volume of elution buffer (50 mM Tris/HCl pH 7.5, 6 M guanidine hydrochloride, 0.3 M imidazole), followed by dialysis against milli-Q water at 4°C. The precipitated multimeric peptide was centrifugated at 8000g, 4°C for 10 min. Sedimented pellets were resuspended in milli-Q water and lyophilized. Samples after each purification step were taken and analyzed by SDS-PAGE. The identity and purity of lyophilized multimeric peptide were determined by MALDI-MS, SDS-PAGE, and RP-HPLC.

### Cleavage of multimeric peptide to AFPpep1 by acidic hydrolysis

To cleave –D–P– bond between AFPpep monomers, several methods were applied [27]. Multimer was dissolved in corresponding hydrolysis buffer (A-L) (Table 1) and left for stirring. Every step sample from the reaction mixture was taken, neutralized by 1 M NH_4_OH, and frozen at −20°C for hydrolysis termination. Then, samples were analyzed by tricine SDS-PAGE.

**Table 1:** Hydrolysis conditions for multimeric peptide cleavage

Method	Hydrolysis conditions	Time, h
A	10% acetic acid, adjusted to pH 2.5 with pyridine, 37°С	48–96
B	10% acetic acid, 25% propanol-1 adjusted to pH 2.5 with pyridine, 37°С	48–96
C	50% formic acid, 37°С	48–96
D	70% formic acid, 37°С	24–96
E	90% formic acid, 37°С	48–72
F	0.1 M HCl + 12%SDS, 37°C	24–120
G	10% acetic acid + 12%SDS, 37°C	24–120
H	0.1 M HCl + 12%SDS, 95°C	0.5–10
I	10% acetic acid + 12%SDS, 95°C	0.5–10
J	Tris-HCl 50 mM pH 7.0 (at RT) + 12%SDS, 95°C	0.5–10
K	10% acetic acid, adjusted to pH 2.5 with pyridine + 6M guanidine HCl, 37°C	24–72
L	0.1 M HCL + 6M guanidine HCl, 37°C	24–72

### Purification of AFPpep1 by RP-HPLC

The hydrolysis mixture (D, 72h) was neutralized with 1 M NH_4_OH to pH 7.0. Peptide sediment was collected by centrifugation at 10,000g for 10 min. Pellets were resuspended in deionized water and lyophilized. The lyophilized mixture was diluted with mobile phase consisted of 0.1% TFA in water (mobile phase A) and pure AcN (mobile phase B) in a ratio of 70:30, v/v. AFPpep1 was purified using 1525 binary pump and 2487 UV–VIS detector (Waters, USA). Separation was performed on Symmetry Prep C18 column with the dimensions of 300 mm × 7.8 mm ID × 7 μm. Isocratic elution at 30% B for 30 min followed by a linear gradient from 30 to 40% B for 20 min and 40 to 70% B for 10 min. The flow rate was kept constant at 3 ml/min. Peaks were detected at 214 nm. The major product peaks were collected, lyophilized, and analyzed by MALDI-MS.

### MALDI-MS

MALDI-MS analysis was performed on UltrafleXtreme TOF/TOF high-resolution mass spectrometer (Bruker Daltonik GmbH, Germany) equipped with Smartbeam II UV laser with following conditions: 10 mg/ml 2,5-dihydroxybenzoic acid solution (20% aqueous AcN, 0.5% TFA) as matrix (0.5 μl per 1.5 μl of the sample), MALDI source in positive mode, analyzer in reflectron mode, and scan range *m*/*z* 500–6500. Spectra were processed with FlexAnalysis 3.3 (Bruker Daltonik GmbH, Germany). The detected *m*/*z* of peptides were compared with the theoretically calculated in accordance with the known amino acid sequences of AFP (NP - 001125.1) and the known genetic sequences of constructs.

### LC-MS

HPLC-ESI-MS analysis was performed on Impact II QqTOF high-resolution mass spectrometer (Bruker Daltonik, Germany) with Elute UHPLC (Bruker Daltonik, Germany) on Intensity Solo 1.8 C18-2 2.1 × 100 mm 1.8 μm 90 Å reverse-phase column (Bruker Daltonik, Germany) with the following conditions: column flow 0.25 ml/min, gradient elution from 5 to 70% B in 25 min (A: 0.1% formic acid in water, B: 0.1% formic acid in AcN), column temperature 40°C, injection volume 5 μl, ESI source in positive mode, HV capillary at 4.5 kV, spray gas–nitrogen at 2.1 bar, dry gas–nitrogen at 8 l/min 220°C, scan range *m*/*z* 50–2200, 2-Hz scan rate for full scan, automatic MS/MS mode (CID) with dynamic scan rate 2–8 Hz, nitrogen as collision gas, collision energy from 23 eV at *m*/*z* 300 to 65 eV at *m*/*z* >1300, and automatic internal calibration with ESI-L low concentration tuning mix (Agilent Technologies, USA). Spectra were processed with BioPharma Compass 3.1.1 (Bruker Daltonik, Germany).

### Cell binding and internalization inhibition assay

Human mammary gland adenocarcinoma cells (MCF-7) were maintained in DMEM medium supplemented with 10% fetal bovine serum and gentamycin (50 μg/ml) in a CO_2_ incubator at 37°C in a humidified atmosphere containing 5% CO_2_. Cells were replated with Trypsin-EDTA solution twice per week. Before the experiment, cells were incubated for 2 h in serum-free DMEM. The concurrent binding and internalization with fluorescein-labeled AFP 3rd domain (3dAFP-FITC) was accessed in order to analyze the AFPpep1 functional activity. The cells were incubated in the presence of 18.5 μM 3dAFP-FITC during 1 h at 4°C or 37°C. AFPpep1 (0.182 mM or 0.281 mM) and not labeled 3dAFP (18.5 μM or 37 μM) were used to interfere with 3dAFP-FITC interaction with AFP receptors. Different concentrations were used to confirm that the inhibition is dose dependent in chosen diapason. A self-inhibition test of 3dAFP-FITC with unlabeled 3dAFP-FITC was used as a control to confirm that this assay works properly. Different temperatures were chosen to find out whether AFPpep1 may inhibit binding to the cell surface or active endocytosis which is in part restricted at +4C. The primary stock solutions of peptides were prepared in DMSO followed by PBS dilution; low-intensity ultrasound was applied if necessary. The fluorescence intensity of cells was measured with a Dako CyAn ADP 9 flow cytometer equipped with an argon laser (488ex nm, 525 nm FITC band-pass). For each sample (2 × 10^3^ cells), the median fluorescence intensity (MFI) was determined. Unstained cells were used as control.

## Results

### Pentameric peptide expression improves yield

**Fig. 1:**
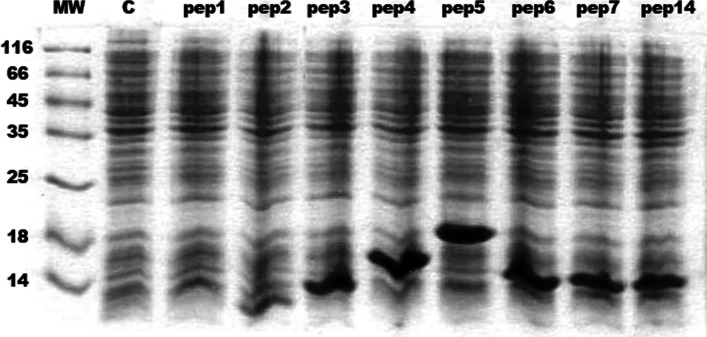
SDS-PAGE of IPTG induced *E. coli* BL21(DE3) transformed with 1–7 and 14 gene repeats (pep1-pep7, pep14), biomass samples. Control—untransformed cells. Bacteria transformed with 8–13 repeats behaved similar to pep7 and pep14 samples on SDS-PAGE (unpublished data)

Recombinant expression of short polypeptides tends to be complicated mostly due to the low proteolytic stability in *E. coli*. All our tries to express monomeric AFP fragment PWGVALQTMKQEFLINLVKQKPQITD (AFPpep1) were unsuccessful. Multimerization facilitated aggregation of the peptide into inclusion bodies. The expression level was proportional to the degree of multimerization, but increased only to 5 tandem repeats (Fig. 1). Transformation with higher than 5 multimeric sequences led to the expression of major product peptide with MW close to pep3 in all cases, probably due to the low intracellular stability of repeated constructs [5]. For further experiments, strain transformed with pentamer (AFPpep5) was used because of the highest stable productivity.

### Purification and hydrolysis of multimer

**Fig. 2:**
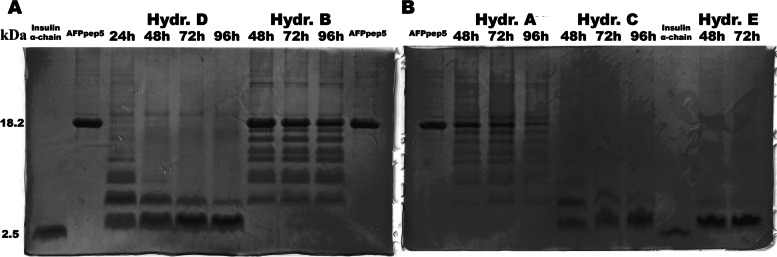
Tricine SDS-PAGE of selected hydrolysis mixtures

For purification purposes, a cleavable his-tag sequence was inserted at C-end. Standard Ni^2+^ chromatography followed by low ionic strength precipitation led to approximately 75% yield of purified pentamer. All of the peptide repeats and his-tag were divided with –D–P– linker. Several mild acidic conditions listed in Table 1 are tested to selectively hydrolyze –D–P– bonds. Based on tricine SDS-PAGE (selected samples, Fig. 2; other, Supplementary information Fig. S2), optimal cleavage with close to 100% conversion and selective formation of ≈ 3 kDa fraction was with either 50–90% formic acid or in SDS-containing tris and HCl solutions.

### Monomer purification and characterization

**Fig. 3:**
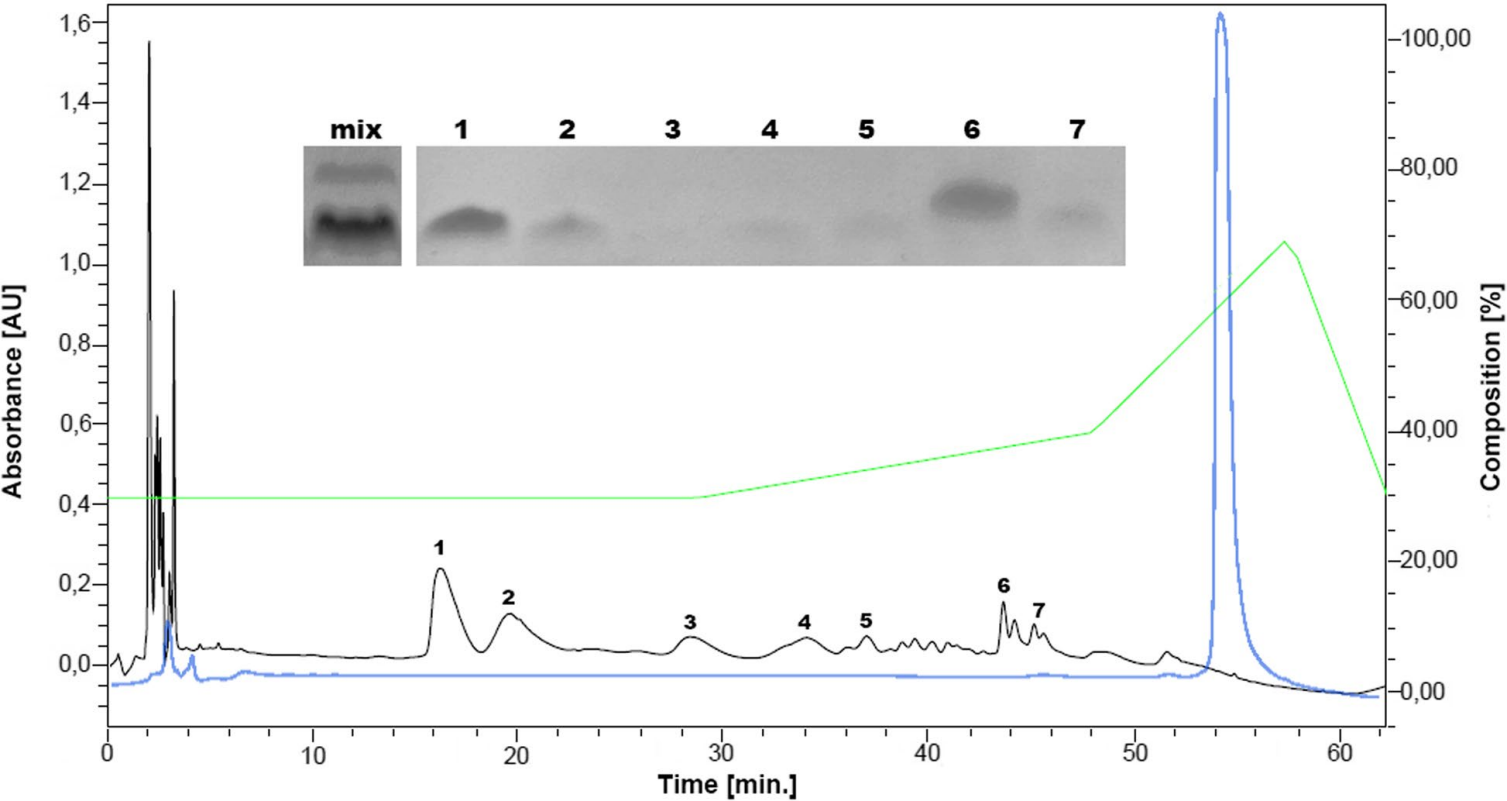
RP-HPLC separation of hydrolysis mixture (Symmetry Prep™ C18 7μm 7.8 × 300 mm). Mobile phase A—0.1% TFA in water, mobile phase B—100% AcN. 30% B isocratic followed by gradient elution from 30 to 70% AcN. Constant flow rate—3 ml/min. Detection—214 nm. The black line—hydrolysis mixture, the blue line—purified AFPpep5 multimer, and the green line—mobile phase composition

**Fig. 4:**
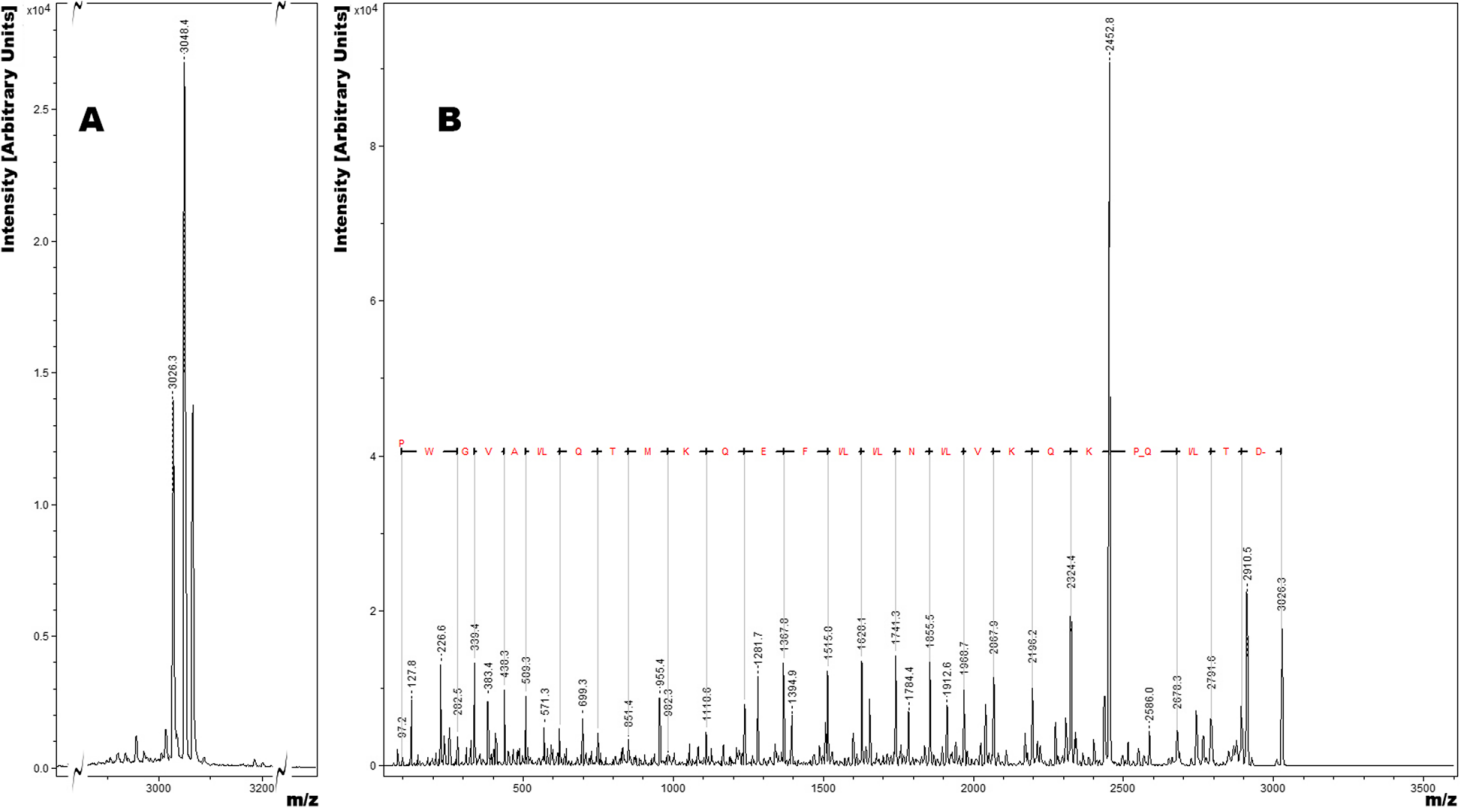
MALDI-MS of AFPpep1 (**A**) and its fragmentation (**B**)

**Fig. 5:**
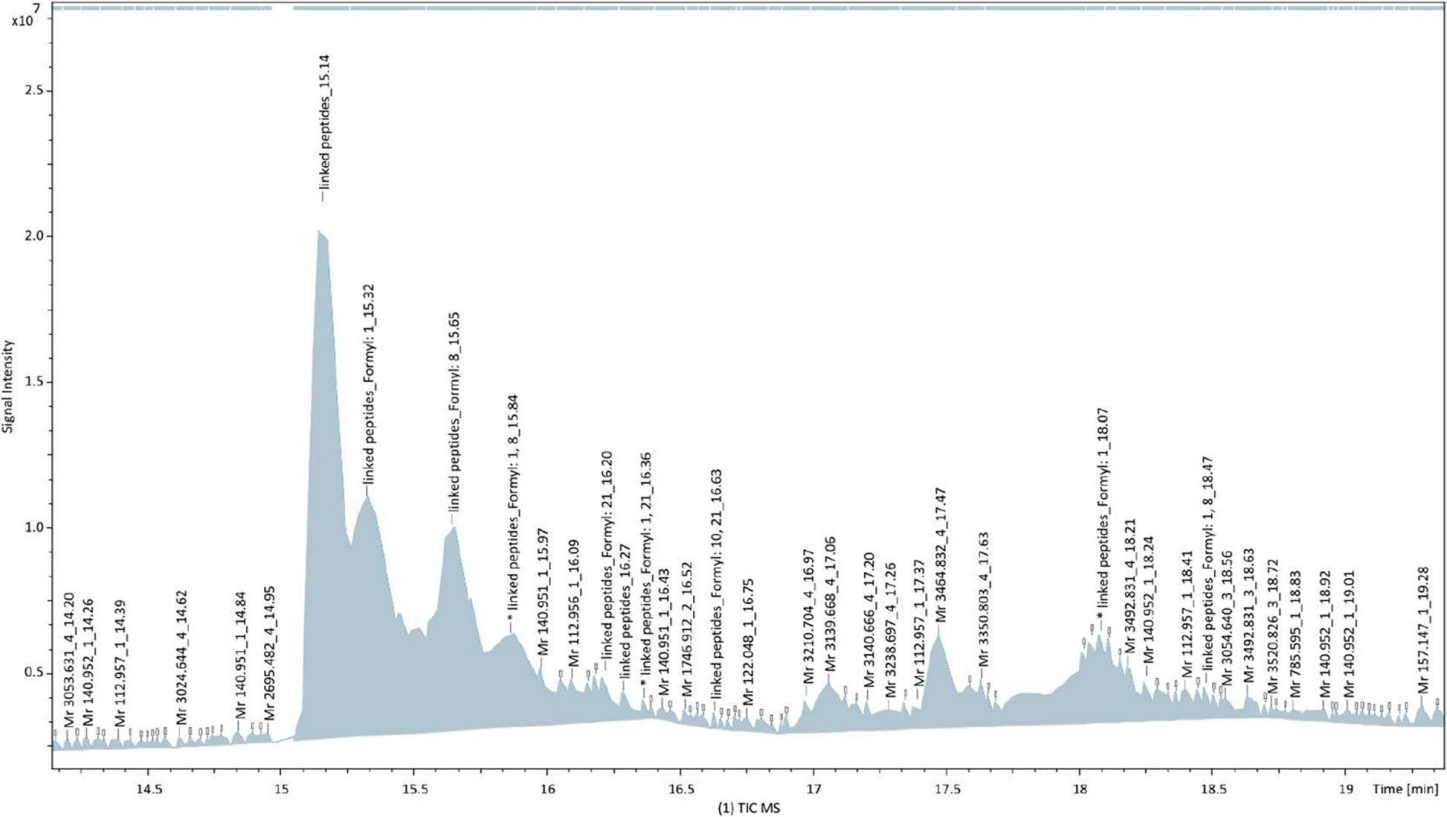
HPLC-ESI-MS of hydrolysis mixture (Intensity Solo 1.8 C18-2 2.1 × 100 mm 1.8 μm 90 Å). Gradient elution from 5 to 70% B in 25 min (A: 0.1% formic acid solution in water, B: 0.1% formic acid solution in AcN). Constant flow rate—0.25 ml/min. Complete product characteristics are given in Supplementary information (Table S1)

AFPpep1 was purified from the formic acid hydrolysis mixture by semi-preparative RP-HPLC. Despite the fact that the majority of peptides in the hydrolysis mixture had similar molecular weight (Fig. 2, Supplementary information Fig. S3), 7 main fractions with different retention times were isolated (Fig. 3). The congruence of peak 1 (Fig. 3) to the AFPpep1 sequence was confirmed by MALDI-MS analysis (Fig. 4). On the first spectrum (Fig. 4A), only 3 peaks were detected, corresponding to AFPpep1 (3026), its Na+ (+22Da), and K+ (+38Da) salts. On the fragmentation spectrum (Fig. 4B), the declared sequence was completely assembled. The closest difficult-to-separate impurity was the monoformylated (+28 Da) derivative AFPpep1 (peak 2, Fig. 3). Dimer, its mono- and di-formylated derivatives, and products of incomplete hydrolysis (AFPpep1-PLDD, AFPpep1-PLEHHHHHHH), as well as products of less specific hydrolysis at W–G, T–D, and Q–T bonds, were detected in the reaction mixture but not in the first two peaks. MALDI-MS failed to obtain the fragmentation spectrum of the formylated derivatives of AFPpep1. However, to the best of our knowledge, previously not detected direct formylation at the N-terminal Pro was observed in the cleaved fragment of the C-end containing affinity tag - PLEHHHHH (Supplementary information Fig. S4). Subsequently, the formylated derivatives of AFPpep1 were determined by HPLC-ESI-MS (Fig. 5). The most significant formylation sites were found to be the N-terminal Pro, similar to the PLEHHHHH fragment, and Tyr8 (O-formylation). N-formylation at Lys10, Lys19, and Lys21 was also detected, but to a much lesser extent. This selectivity of formylation may be the result of kinetic or steric differences. It is possible that formylation of the imino group of N-terminal proline may be specifically coupled to cleavage reaction.

### Cell binding and internalization

**Fig. 6:**
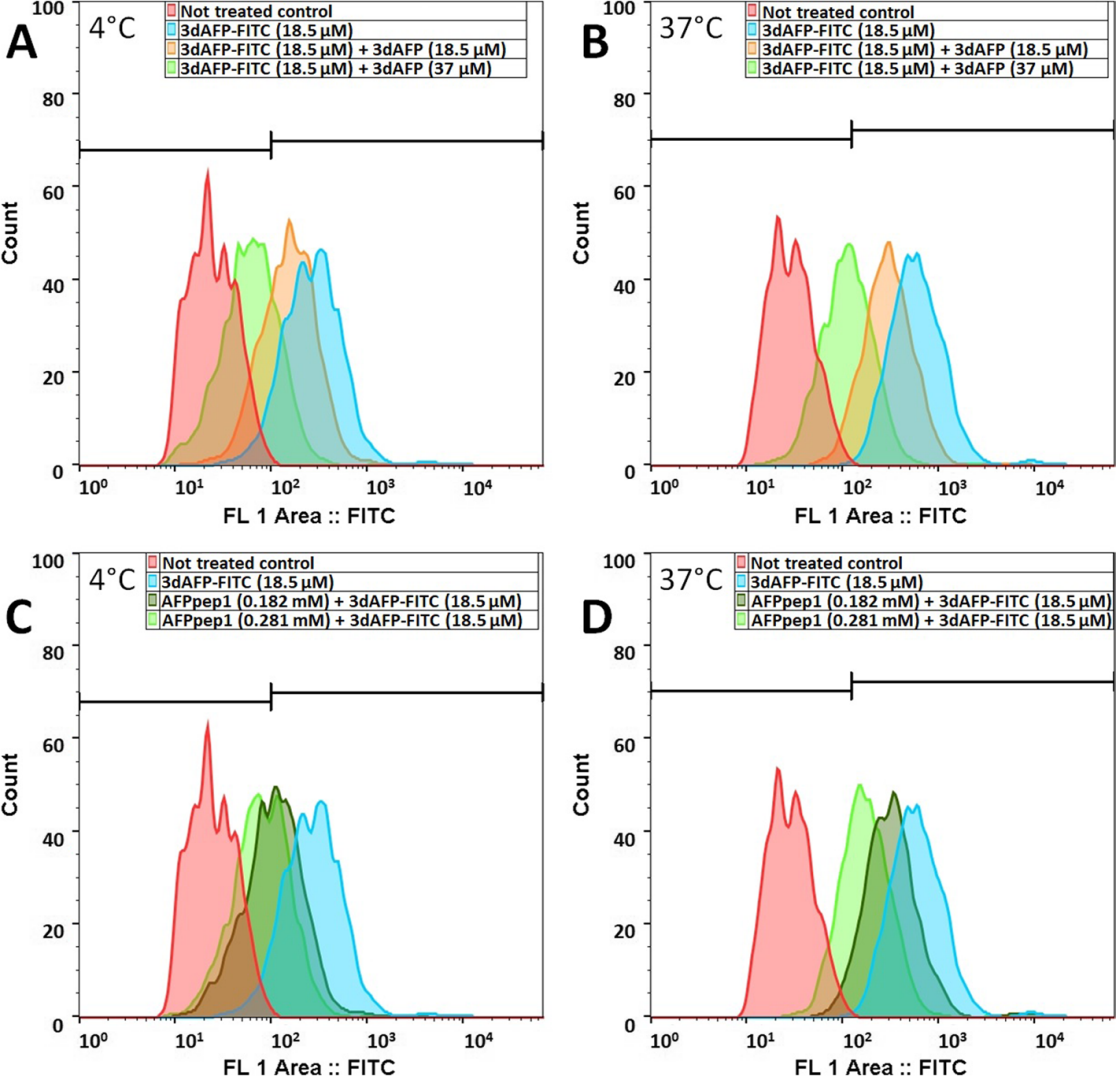
Concurrent cell binding (**A**, **C**) and internalization assay (**B**, **D**). Auto-inhibition of 3dAFP-FITC binding (**A**) and internalization (**B**) by unlabeled 3dAFP. Inhibition of 3dAFP-FITC binding (**C**) and internalization (**D**) by unlabeled AFPpep1. Unstained cells were used as the control (red). Flow cytometry, MCF-7 cells

Cell binding and internalization ability of purified AFPpep1 was accessed through inhibition of complete AFP 3rd domain (3dAFP) described previously [26]. Self-inhibition of cell binding and internalization with labeled and unlabeled 3dAFP was performed as a control (Fig. 6A, B). Co-incubation of AFPpep1 with labeled 3dAFP led to a decrease of fluorescent signal due to concurrent binding (Fig. 6C) and internalization (Fig. 6D). Comparable inhibition of cell binding and internalization was observed only with a high molar excess of the peptide, which is probably due to the greater affinity of the large protein fragment. On the other hand, limited inhibition may result from more specific binding of the peptide to the canonical AFP receptor.

## Discussion

Sequential multimerization of the AFP peptide fragment from 1 to 5 repeats led to a gradual increase in the stability of the expression product, while multimers containing 6 or more repeats were not detected by SDS-PAGE. Thus, the expression of multimers tends to be possible up to a certain number of repeats, which is a sequence-specific value. For the selected AFP fragment, the pentamer turned out to be the largest in size among the stable ones and had the highest level of expression. Various methods of the multimer hydrolysis at –D–P– bonds were analyzed; however, we were not lucky to overperform the known standard method with formic acid. Multimer solubility in hydrolysis buffer seems to be the most critical parameter for optimal –D–P– cleavage while formic acid is best suited for dissolving almost any peptide. Despite the fact that SDS-containing hydrolysis samples had bands with characteristic MW of hydrolysis fractions, we did not find any possible way to completely separate peptides from SDS, which may interfere with mass spectrometry. MALDI-MS confirmed the presence of monomeric AFPpep1 (Supplementary information Fig. S3) only in formic acid cleaved mixtures (D, G, and J conditions were tested). We assume that if a method for separation of peptides from SDS was available, tris-buffered hydrolysis with SDS would provide a convenient alternative to formic acid. Hydrolysis in tris buffer can be easily controlled by temperature (as the temperature rises, pH shifts to the acidic) and does not require neutralization for termination. Also, side reactions with the formation of formyl derivatives would be excluded. After hydrolysis with formic acid, we managed to find a simple one-step semi-preparative RP-HPLC method for separating the target peptide from formylated and less specific hydrolysis products. It is important to note that of all the formylation sites, only Lys10 is part of Moro’s minimal receptor-binding peptide [28]. The low degree of formylation at this site and the possibility of preparative separation (Fig. 3) will minimize the risks of loss of the receptor-binding activity of this peptide. The purified peptide structure was fully assembled by tandem mass spectrometry (Fig. 4), and the activity was confirmed by competitive inhibition of binding and internalization of the larger fluorescently labeled AFP fragment (Fig. 6).

## Conclusions

Multimeric expression of peptides can be performed with –D–P– bond linkers. Such technique reveals a high peptide expression level with localization in inclusion bodies, which supports cleavable purification tags insert. Gradually, with an increase in multimerization, the number of expressed repeats stabilizes; in our case, it stopped at 5. Probably, larger fragments are also expressed in insignificant undetectable amounts, but we did not find any evidence for that [29]. Cleavage of as high as 10 linkers in one construct can be done with up to 100% conversion and high selectivity using optimized conditions in formic acid. The target monomeric product was represented by the largest peak on both analytical and semi-preparative HPLC and can be separated from close formyl-Pro, formyl-Tyr, and formyl-Lys derivatives. New formic acid-free hydrolysis methods still could prove even higher selectivity if other easily removable solubilization agents were found. Perhaps, development of selective deformylation methods could also contribute to the improvement of this technique. In this work, a method is described that allows one to obtain a recombinant AFP fragment in semi-preparative amounts in the highest quality and quantity and in the very efficient way. This fragment can find application in cytotoxic drug delivery [18, 24, 30] or as an inhibitor of endogenous AFP; however, more research is still to be done. The reported procedure is characterized by the lower reagent cost in comparison with enzymatic hydrolysis of peptide fusions or solid-phase synthesis and may be adopted for different peptide expression, especially with low amino and hydroxy side chain content.

## Supplementary Information

**Additional file 1: **Supplementary information. **Figure S1.** Cloning strategy. **Figure S2.** Tricine-SDS-PAGE analysis of different hydrolysis methods. **Figure S3.** Analysis of hydrolysis mixture D – 96h by MALDI-MS (3026.2 – AFPpep1; 3082.2 – formyl-AFPpep1). **Figure S4.** Fragmentation spectrum of formyl-PLEHHHHH. **Table S1.** Formic acid hydrolysis products by HPLC-ESI-MS.

**Additional file 2.** Supplementary information 2. Reagents.

## Data Availability

All the data generated and/or analyzed during this study is included in this published article.

## Abbreviations

(U)HPLC: (Ultra) high-performance liquid chromatography
3dAFP: Alpha-fetoprotein (human) third (C-end) domain
AcN: Acetonitrile
AFP: Alpha-fetoprotein (human)
AFPpep1: PWGVALQTMKQEFLINLVKQKPQITD
AFPpep5: MADPWGVALQTMKQEFLINLVKQKPQITDPLDDPWGVALQTMKQEFLINLVKQKPQITDPLDDPWGVALQTMKQEFLINLVKQKPQITDPLDDPWGVALQTMKQEFLINLVKQKPQITDPLDDPWGVALQTMKQEFLINLVKQKPQITDPLEHHHHH
BCA: Bicinchoninic acid
CID: Collision-induced dissociation
DMEM: Dulbecco’s modified Eagle medium
DMSO: Dimethylsulfoxide
EDTA: Ethylenediaminetetraacetic acid
ESI: Electrospray ionization
FBS: Fetal bovine serum
FITC: Fluorescein isothiocyanate
HV: High voltage
IPTG: Isopropylthio-β-galactoside
LB: Luria-Bertani medium
MALDI: Matrix-assisted laser desorption ionization
MCF-7: Human mammary gland adenocarcinoma cells
MFI: Mean fluorescence intensity
MS: Mass spectrometry
PAGE: Polyacrylamide gel electrophoresis
PCR: Polymerase chain reaction
RP: Reverse phase
SDS: Sodium dodecyl sulfate
TFA: Trifluoroacetic acid
TOF: Time of flight
UV–VIS: Ultraviolet–visible absorption
