# Supplementary material for: Expression of acid cleavable Asp-Pro linked multimeric AFP peptide in E. coli
Source: J Genet Eng Biotechnol. 2021 Oct 14;19:155. doi: 10.1186/s43141-021-00265-5 (PMC8517049; doi:10.1186/s43141-021-00265-5)
Supplement: Supplementary file 1 — Additional file 1: Supplementary information. Figure S1. Cloning strategy. Figure S2. Tricine-SDS-PAGE analysis of different hydrolysis methods. Figure S3. Analysis of hydrolysis mixture D – 96h by MALDI-MS (3026.2 – AFPpep1; 3082.2 – formyl-AFPpep1). Figure S4. Fragmentation spectrum of formyl-PLEHHHHH. Table S1. Formic acid hydrolysis products by HPLC-ESI-MS. [file 43141_2021_265_MOESM1_ESM.docx]

**Supplementary information**

**
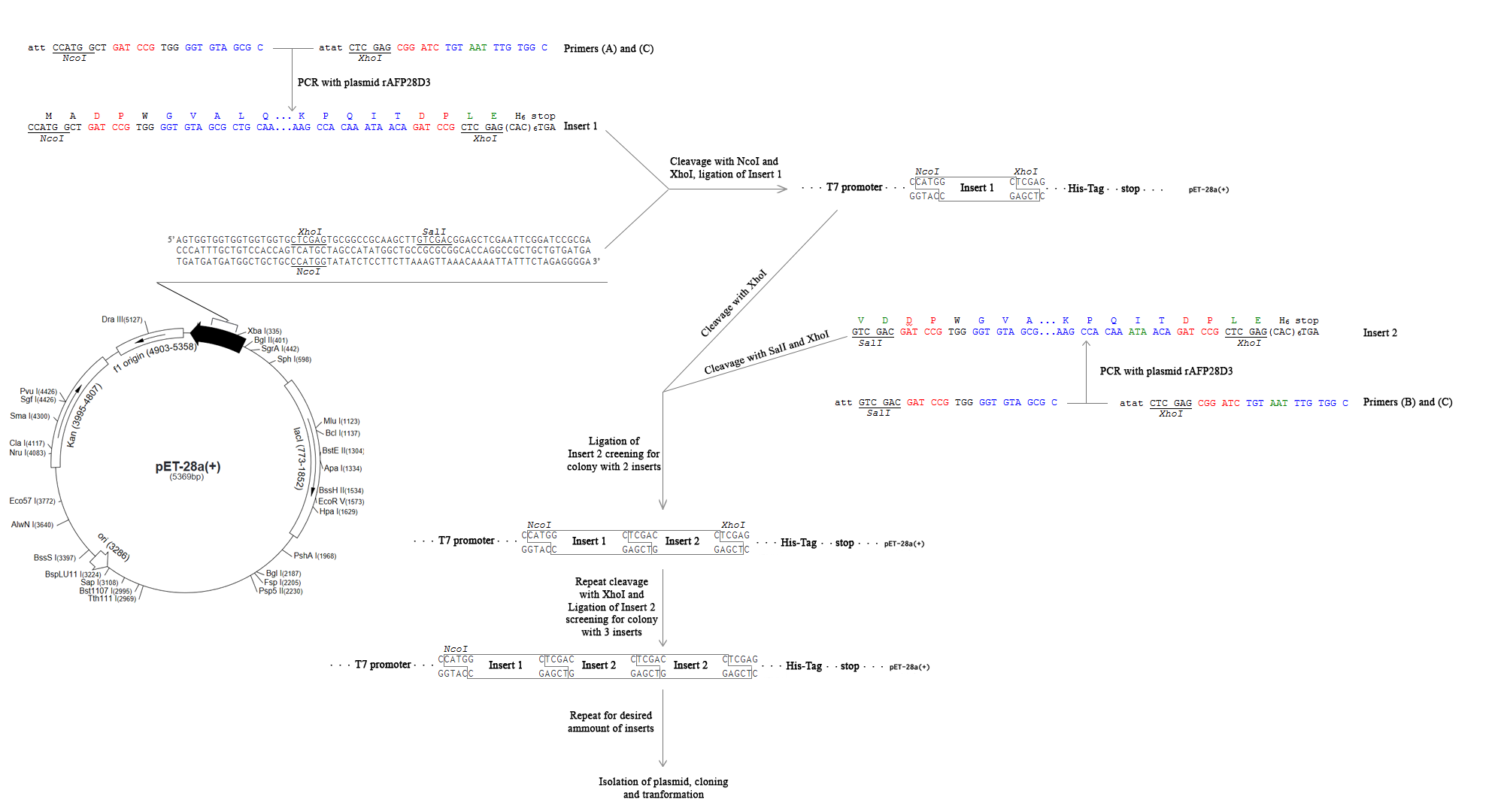
**

**Fig. S1.** Cloning strategy

**
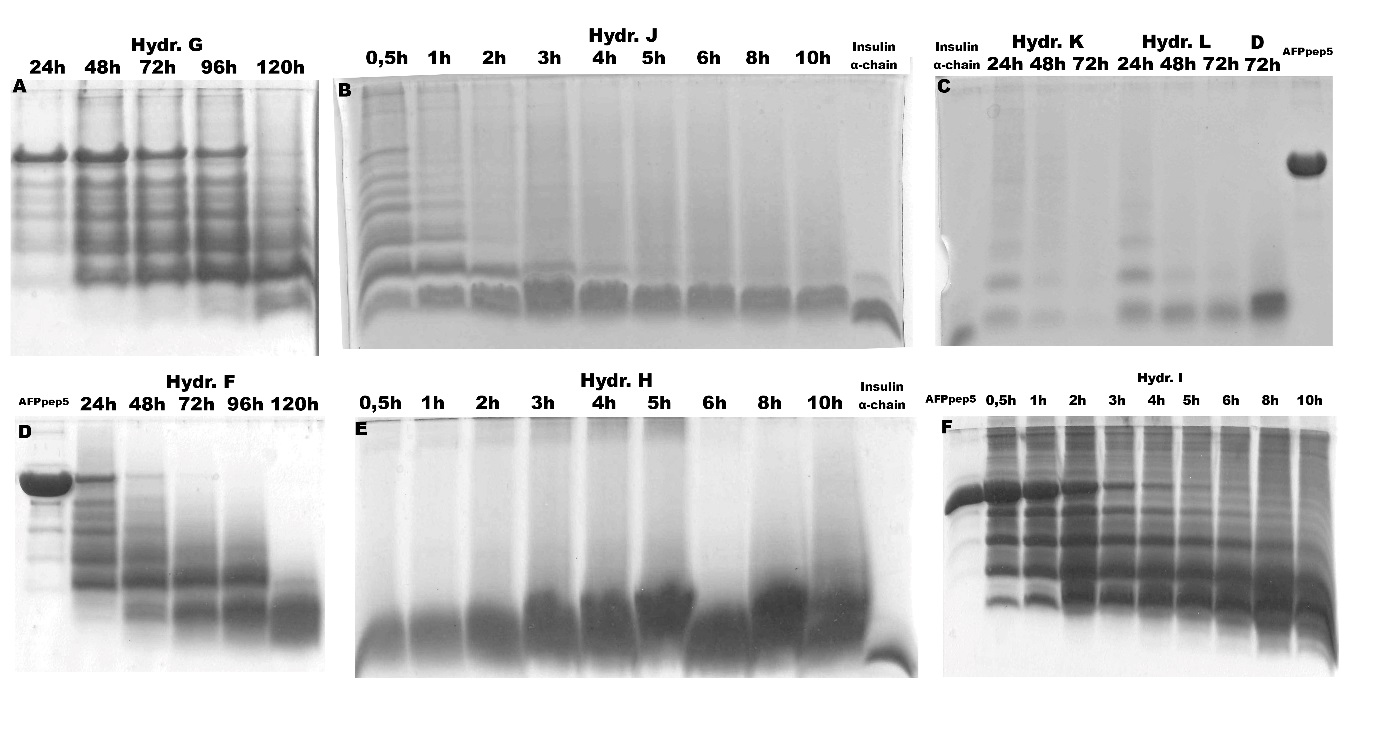
**

**Fig. S2.** Tricine-SDS-PAGE analysis of different hydrolysis methods


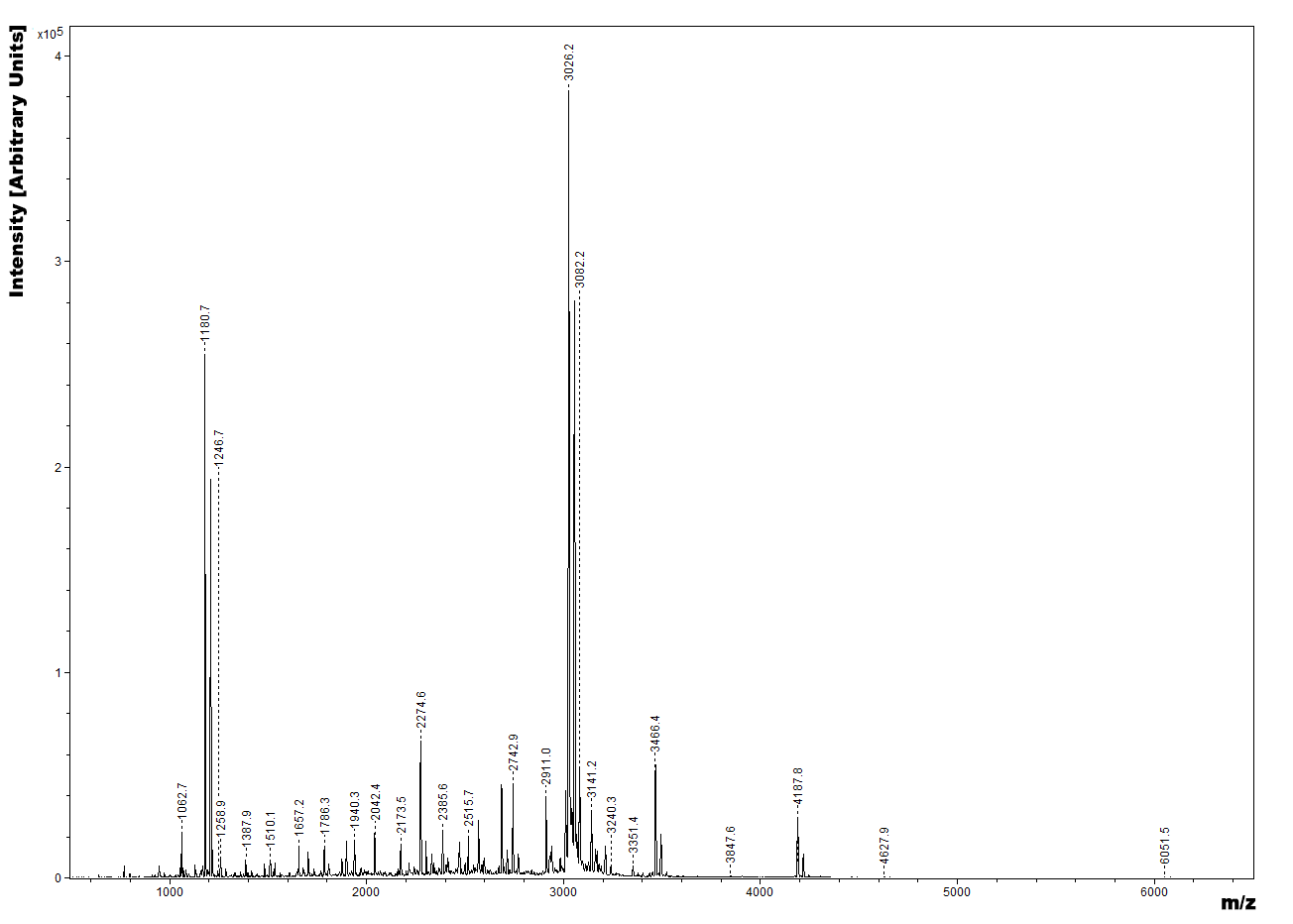


**Fig. S3.** Analysis of hydrolysis mixture D – 96h by MALDI-MS (3026.2 – AFPpep1; 3082.2 – formyl-AFPpep1)

**
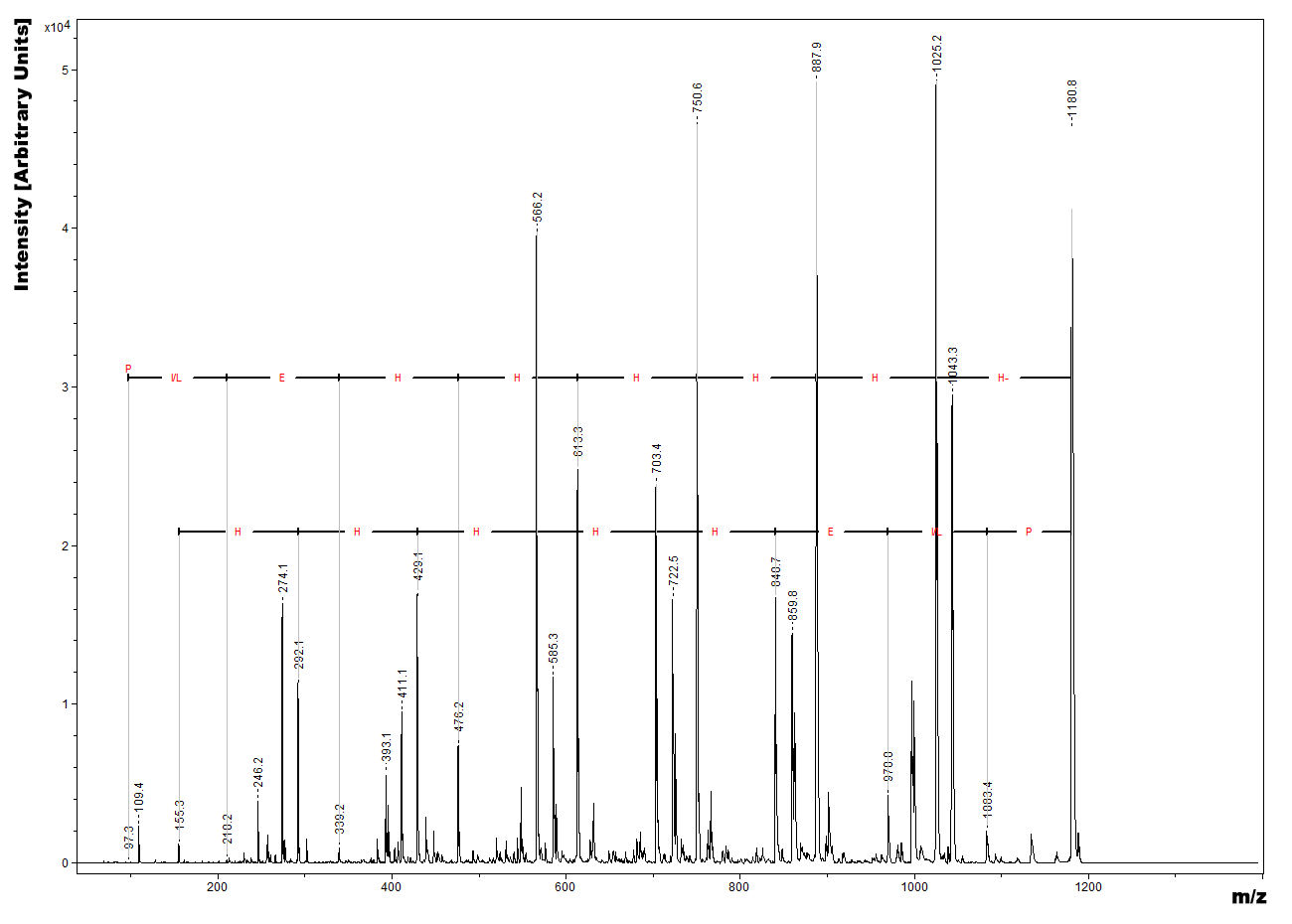
**

**Fig. S4.** Fragmentation spectrum of formyl-PLEHHHHH.

**Table. S1.** Formic acid hydrolysis products by HPLC-ESI-MS

|  | | |  |  | | |  | | |  | |  |  | | |  |
| --- | --- | --- | --- | --- | --- | --- | --- | --- | --- | --- | --- | --- | --- | --- | --- | --- |
| **Rt [min]** | **m/z measured** | | **Mr calculated** | **z** | **Δ m/z [ppm]** | | **Intensity** | **Score** | | **Sequence** | | | **Modifications** |  |  |  |
| 1,11 | 118,0864 | | 117,079 | 1 | 0,88 | | 12 190 | 31 | | L.V.K | | |  |  |  |  |
| 15,02 | 937,9925 | | 1873,976 | 2 | -2,96 | | 3 898 | 16,4 | | -.PWGVALQTMKQEFLIN.L | | |  |  |  |  |
| 15,06 | 618,6003 | | 2470,377 | 4 | -1,95 | | 16 810 | 40,3 | | -.PWGVALQTMKQEFLINLVKQK.P | | |  |  |  |  |
| 15,14 | 605,9355 | | 3024,647 | 5 | -1,92 | | 7 732 | 19,3 | | -.PWGVALQTMKQEFLINLVKQKPQITD.- | | |  |  |  |  |
| 15,14 | 757,1676 | | 3024,647 | 4 | -1,96 | | 1 907 000 | 57,5 | | -.PWGVALQTMKQEFLINLVKQKPQITD.- | | |  |  |  |  |
| 15,17 | 1009,2208 | | 3024,647 | 3 | -2,09 | | 299 500 | 56,8 | | -.PWGVALQTMKQEFLINLVKQKPQITD.- | | |  |  |  |  |
| 15,21 | 728,4109 | | 2909,6201 | 4 | -1,91 | | 96 970 | 63,1 | | -.PWGVALQTMKQEFLINLVKQKPQIT.D | | |  |  |  |  |
| 15,21 | 970,8787 | | 2909,6201 | 3 | -1,97 | | 18 360 | 48,2 | | -.PWGVALQTMKQEFLINLVKQKPQIT.D | | |  |  |  |  |
| 15,21 | 970,8787 | | 2909,6201 | 3 | -1,97 | | 18 360 | 48,2 | | -.PWGVALQTMKQEFLINLVKQKPQIT.D | | |  |  |  |  |
| 15,32 | 764,1659 | | 3052,642 | 4 | -2,5 | | 450 300 | 47 | | -.PWGVALQTMKQEFLINLVKQKPQITD.- | | | Formyl: 1 |  |  |  |
| 15,36 | 735,4093 | | 2937,615 | 4 | -2,42 | | 22 340 | 46,9 | | -.PWGVALQTMKQEFLINLVKQKPQIT.D | | | Formyl: 1 |  |  |  |
| 15,36 | 735,4093 | | 2937,615 | 4 | -2,42 | | 22 340 | 46,9 | | -.PWGVALQTMKQEFLINLVKQKPQIT.D | | | Formyl: 1 |  |  |  |
| 15,65 | 764,166 | | 3052,642 | 4 | -2,27 | | 444 300 | 50,3 | | -.PWGVALQTMKQEFLINLVKQKPQITD.- | | | Formyl: 8 |  |  |  |
| 15,65 | 1018,5522 | | 3052,642 | 3 | -2,34 | | 81 540 | 51,2 | | -.PWGVALQTMKQEFLINLVKQKPQITD.- | | | Formyl: 8 |  |  |  |
| 15,69 | 735,409 | | 2937,615 | 4 | -2,71 | | 20 220 | 40,2 | | -.PWGVALQTMKQEFLINLVKQKPQIT.D | | | Formyl: 10 |  |  |  |
| 15,69 | 980,208 | | 2937,615 | 3 | -4,41 | | 4 866 | 18,8 | | -.PWGVALQTMKQEFLINLVKQKPQIT.D | | | Formyl: 8 |  |  |  |
| 15,69 | 980,208 | | 2937,615 | 3 | -4,41 | | 4 866 | 18,8 | | -.PWGVALQTMKQEFLINLVKQKPQIT.D | | | Formyl: 10 |  |  |  |
| 15,69 | 980,208 | | 2937,615 | 3 | -4,41 | | 4 866 | 18,8 | | -.PWGVALQTMKQEFLINLVKQKPQIT.D | | | Formyl: 8 |  |  |  |
| 15,69 | 980,208 | | 2937,615 | 3 | -4,41 | | 4 866 | 18,8 | | -.PWGVALQTMKQEFLINLVKQKPQIT.D | | | Formyl: 10 |  |  |  |
| 15,81 | 771,1638 | | 3080,6369 | 4 | -3,43 | | 83 200 | 43 | | -.PWGVALQTMKQEFLINLVKQKPQITD.- | | | Formyl: 1, 8 |  |  |  |
| 16,2 | 1018,552 | | 3052,642 | 3 | -2,6 | | 34 540 | 40,7 | | -.PWGVALQTMKQEFLINLVKQKPQITD.- | | | Formyl: 21 |  |  |  |
| 16,2 | 1018,552 | | 3052,642 | 3 | -2,6 | | 34 540 | 44,2 | | -.PWGVALQTMKQEFLINLVKQKPQITD.- | | | Formyl: 19 |  |  |  |
| 16,27 | 739,0802 | | 2214,2235 | 3 | -2,12 | | 9 206 | 50,4 | | -.PWGVALQTMKQEFLINLVK.Q | | |  |  |  |  |
| 16,36 | 1027,8834 | | 3080,6369 | 3 | -2,71 | | 8 196 | 15,1 | | -.PWGVALQTMKQEFLINLVKQKPQITD.- | | | Formyl: 1, 21 |  |  |  |
| 16,63 | 1027,883 | | 3080,6369 | 3 | -3,1 | | 7 936 | 20,6 | | -.PWGVALQTMKQEFLINLVKQKPQITD.- | | | Formyl: 8, 19 |  |  |  |
| 16,63 | 1027,883 | | 3080,6369 | 3 | -3,1 | | 7 936 | 17,9 | | -.PWGVALQTMKQEFLINLVKQKPQITD.- | | | Formyl: 10, 21 |  |  |  |
| 18,04 | 1018,5544 | | 3052,642 | 3 | -0,15 | | 167 400 | 47 | | -.PWGVALQTMKQEFLINLVKQKPQITD.- | | | Formyl: 10 |  |  |  |
| 18,04 | 1018,5544 | | 3052,642 | 3 | -0,15 | | 167 400 | 59,6 | | -.PWGVALQTMKQEFLINLVKQKPQITD.- | | | Formyl: 1 |  |  |  |
| 18,07 | 980,2118 | | 2937,615 | 3 | -0,49 | | 11 460 | 25 | | -.PWGVALQTMKQEFLINLVKQKPQIT.D | | | Formyl: 1 |  |  |  |
| 18,07 | 980,2118 | | 2937,615 | 3 | -0,49 | | 11 460 | 25 | | -.PWGVALQTMKQEFLINLVKQKPQIT.D | | | Formyl: 1 |  |  |  |
| 18,47 | 1027,8858 | | 3080,6369 | 3 | -0,42 | | 39 580 | 38 | | -.PWGVALQTMKQEFLINLVKQKPQITD.- | | | Formyl: 1, 10 |  |  |  |
| 18,47 | 1027,8858 | | 3080,6369 | 3 | -0,42 | | 39 580 | 54,8 | | -.PWGVALQTMKQEFLINLVKQKPQITD.- | | | Formyl: 1, 8 |  |  |  |
