# Supplementary material for: Expression of acid cleavable Asp-Pro linked multimeric AFP peptide in E. coli
Source: J Genet Eng Biotechnol. 2021 Oct 14;19:155. doi: 10.1186/s43141-021-00265-5 (PMC8517049; doi:10.1186/s43141-021-00265-5)
Supplement: Supplementary file 2 — Additional file 2. Supplementary information 2. Reagents. [file 43141_2021_265_MOESM2_ESM.docx]

**Supplementary information 2**

**Reagents**

BCA kit, FITC, trizma base, trizma-HCl, nickel chloride (Sigma-Aldrich, USA); imidazole (Acros, USA); tryptone, yeast extract and agar (Becton, Dickinson and Company, USA), IPTG (Helicon, Russia), ethanol (Merck Millipore, Billerica, MA, USA); NcoI, SalI, XhoI restrictases, T4 phage polynucleotide kinase, T4 phage DNA ligase, related buffers and PCR kit from Thermo Fisher Scientific Inc. (Waltham, MA, USA); Quantum Prep Plasmid Miniprep Kit (BIO-RAD, USA); DMEM (Gibco, USA); sodium dodecyl sulfate (Merck, Germany); guanidine hydrochloride (Helicon, Russia); acetic acid (Sigma-Aldrich, USA); hydrochloric acid (Chimmed, Russia); propanol-1 (Sigma-Aldrich, USA); pyridine (Fluka AG, Switzerland); formic acid (Sigma-Aldrich, USA) and 98 - 100% BioChemica (PanReac, Germany); trifluoroacetic acid (Sigma-Aldrich, USA); 2,5-Dihydroxybenzoic acid (Sigma-Aldrich, USA); acetonitrile: LiChrosolv hypergrade for LC-MS (Supelco, Germany); water: type 1, Aqualab AL-4 (MedianaFilter, Russia), DMEM and FBS (Gibco, USA), gentamycin (Paneco, Russia), FITC (Sigma-Aldrich, USA).
